# Mapping brain morphology to cognitive deficits: a study on PD-CRS scores in Parkinson’s disease with mild cognitive impairment

**DOI:** 10.3389/fnana.2024.1362165

**Published:** 2024-08-14

**Authors:** Pedro Renato Brandão, Danilo Assis Pereira, Talyta Cortez Grippe, Diógenes Diego de Carvalho Bispo, Fernando Bisinoto Maluf, Ricardo Titze-de-Almeida, Brenda Macedo de Almeida e Castro, Renato Puppi Munhoz, Maria Clotilde Henriques Tavares, Francisco Cardoso

**Affiliations:** ^1^Neuroscience and Behavior Lab, Biological Sciences Institute, University of Brasília (UnB), Brasília, Brazil; ^2^Hospital Sírio-Libanês, Instituto de Ensino e Pesquisa, Brasília, Brazil; ^3^Brazilian Institute of Neuropsychology and Cognitive Sciences (IBNeuro), Brasília, Brazil; ^4^Movement Disorders Centre, Toronto Western Hospital, University of Toronto, Toronto, ON, Canada; ^5^Radiology Department, Brasilia University Hospital (HUB-UnB), University of Brasília (UnB), Brasília, Brazil; ^6^Radiology Department, Santa Marta Hospital, Taguatinga, Brazil; ^7^Central Institute of Sciences, Research Center for Major Themes – Neurodegenerative disorders, University of Brasília, Brasília, Brazil; ^8^Internal Medicine, Neurology Service, Movement Disorder Centre, The Federal University of Minas Gerais, Belo Horizonte, Brazil

**Keywords:** Parkinson’s Disease-Cognitive Rating Scale, mild cognitive impairment, cortical volume, cortical thickness, magnetic resonance imaging, Parkinson’s disease, neuroimaging

## Abstract

**Background:**

The Parkinson’s Disease-Cognitive Rating Scale (PD-CRS) is a widely used tool for detecting mild cognitive impairment (MCI) in Parkinson’s Disease (PD) patients, however, the neuroanatomical underpinnings of this test’s outcomes require clarification. This study aims to: (a) investigate cortical volume (CVol) and cortical thickness (CTh) disparities between PD patients exhibiting mild cognitive impairment (PD-MCI) and those with preserved cognitive abilities (PD-IC); and (b) identify the structural correlates in magnetic resonance imaging (MRI) of overall PD-CRS performance, including its subtest scores, within a non-demented PD cohort.

**Materials and methods:**

This study involved 51 PD patients with Hoehn & Yahr stages I–II, categorized into two groups: PD-IC (*n* = 36) and PD-MCI (*n* = 15). Cognitive screening evaluations utilized the PD-CRS and the Montreal Cognitive Assessment (MoCA). PD-MCI classification adhered to the Movement Disorder Society Task Force criteria, incorporating extensive neuropsychological assessments. The interrelation between brain morphology and cognitive performance was determined using FreeSurfer.

**Results:**

Vertex-wise analysis of the entire brain demonstrated a notable reduction in CVol within a 2,934 mm^2^ cluster, encompassing parietal and temporal regions, in the PD-MCI group relative to the PD-IC group. Lower PD-CRS total scores correlated with decreased CVol in the middle frontal, superior temporal, inferior parietal, and cingulate cortices. The PD-CRS subtests for Sustained Attention and Clock Drawing were associated with cortical thinning in distinct regions: the Clock Drawing subtest correlated with changes in the parietal lobe, insula, and superior temporal cortex morphology; while the PD-CRS frontal-subcortical scores presented positive correlations with CTh in the transverse temporal, medial orbitofrontal, superior temporal, precuneus, fusiform, and supramarginal regions. Additionally, PD-CRS subtests for Semantic and Alternating verbal fluency were linked to CTh changes in orbitofrontal, temporal, fusiform, insula, and precentral regions.

**Conclusion:**

PD-CRS performance mirrors neuroanatomical changes across extensive fronto-temporo-parietal areas, covering both lateral and medial cortical surfaces, in PD patients without dementia. The observed changes in CVol and CTh associated with this cognitive screening tool suggest their potential as surrogate markers for cognitive decline in PD. These findings warrant further exploration and validation in multicenter studies involving independent patient cohorts.

## Background

Cognitive deterioration and the onset of dementia stand as challenging facets related to the progression of Parkinson’s Disease (PD), substantially amplifying the overall disease burden of disability (Emre et al., 2014; Aarsland et al., 2017; Obeso et al., 2017; Brandão et al., 2020). The prevalence of dementia associated with PD (PDD) is particularly overwhelming, with an estimate indicating that it affects over 80% of patients two decades after the onset of the disease (Hely et al., 2008). Although the understanding of the natural history of PDD evolved significantly, many challenges persist, including the development of adequate rating scales for accurate phenotyping and the identification of reliable biomarkers, both fluid-and imaging-based, for disease staging (Aarsland et al., 2021; Cardoso et al., 2024). Addressing these needs would not only allow correlation between biomarkers, clinical and cognitive manifestations but also facilitate the early identification of individuals at elevated risk for accelerated cognitive decline (Brandão et al., 2020).

The spectrum of cognitive deficits in PD ranges from subtle disturbances in newly diagnosed patients to severe dysfunction in those with PDD. Bridging these extremes is mild cognitive impairment (MCI), a construct defined by cognitive-related complaints and objective cognitive deficits in the absence of significant functional impairment (Aarsland et al., 2010; Litvan et al., 2012; Pedersen et al., 2017). PD-MCI represents a window for understanding the trajectory of cognitive decline and identifying potential biomarkers and therapeutic targets. The Movement Disorder Society (MDS) Task Force has proposed diagnostic criteria for PD-MCI, which can be assessed using either abbreviated (Level I) or comprehensive (Level II) neuropsychological assessment (Litvan et al., 2012). The Parkinson’s Disease-Cognitive Rating Scale (PD-CRS) has emerged as a widely used tool demonstrating good discriminative validity for PD-MCI (Pagonabarraga et al., 2008; Brandão et al., 2023).

Advances in neuroimaging have provided insights into the structural brain changes associated with cognitive impairment in PD. Studies have revealed patterns of cortical thinning and atrophy in PD-MCI, particularly in posterior cortical regions. In patients with PD who exhibit mild cognitive impairment (PD-MCI), a distinct pattern of localized cortical thickness (CTh) reduction has been consistently observed (Hanganu et al., 2014; Segura et al., 2014). This cortical thinning is primarily evident in areas associated with cortical and limbic degeneration, including, but not limited to, the mesial temporal lobes and posterior medial cortical regions (Pagonabarraga et al., 2013). Further, empirical studies have demonstrated correlations between these structural brain abnormalities and cognitive performance metrics. For example, diminished performance in specific tasks of the Mini-Mental State Examination (MMSE), such as the pentagon copying task, correlates with regional atrophy in the temporal–parietal-occipital cortex (Garcia-Diaz et al., 2014). Similarly, cognitive deficits assessed by the Montreal Cognitive Assessment (MoCA) are associated with cortical thinning in various frontal and temporoparietal regions, notably the superior frontal and inferior parietal lobules, as well as the orbitofrontal cortex, fusiform gyrus, and parahippocampal gyrus (Mak et al., 2015).

Nonetheless, the neuroanatomical correlates of the PD-CRS (Pagonabarraga et al., 2008; Brandão et al., 2023)—a global assessment tool recommended by MDS for screening both dementia and mild cognitive impairment (Skorvanek et al., 2018)—remain underexplored. Prior research focusing on PD-MCI has tried to correlate performance in PD-CRS subtests and imaging data: for example, immediate verbal free-recall was found to correlate with thinning in the cuneus region, whereas delayed free-recall exhibited associations with cortical thinning in the anteromedial temporal cortex, parahippocampus, and lingual gyrus (Pagonabarraga et al., 2013). Furthermore, in the context of the PD-CRS’s posterior cortical tasks, a discernible relationship was observed between figure naming and reduced cortical thickness in the fusiform gyrus, temporal pole, and parahippocampus. Similarly, impairments in the clock-copying task were linked to a reduction in CTh in the precuneus, parahippocampus, and lingual gyrus in PDD.

The present study was designed to investigate the relationship between brain morphology and cognitive performance as assessed by the PD-CRS in a sample of non-demented PD patients, with two specific objectives. The first was to examine changes in CTh and cortical volume (CVol) among PD patients exhibiting mild cognitive impairment (PD-MCI), in comparison to a control group of PD patients with intact cognition (PD-IC). This investigation employed high-field magnetic resonance imaging (MRI) techniques. The second objective focused on identifying regional brain substrates associated with performance metrics on the PD-CRS. This aspect of the study involved a cohort of PD patients without dementia and was aimed at furthering our understanding of the brain-behavior relationships captured by PD-CRS, and inform future efforts to parse the heterogeneity of cognitive deficits in PD.

## Methods

### Ethical compliance statement

This study was conducted in accordance with the ethical principles outlined in the Declaration of Helsinki and its subsequent amendments. The research protocol was reviewed and approved by the Research Ethics Committee of the Centro Universitário de Brasília (CEUB) under the reference number 07073419.0.0000.0023. Prior to their participation, all subjects provided written informed consent.

### Study design

This study was designed as a prospective, analytical, and cross-sectional investigation. The recruitment of participants began in the first half of 2019 and was completed by August 2021. Notably, the enrollment process was temporarily halted for the majority of 2020 as a precautionary measure against the spread of SARS-CoV-2 during the COVID-19 pandemic. This decision was made to ensure the safety of participants and to adhere to public health guidelines. The primary research activities were coordinated at the Neuroscience and Behavior Laboratory, located at the Darcy Ribeiro Campus of the University of Brasília, Brasília, Brazil.

### Participants

Patients with PD were recruited from the community through a comprehensive approach. This included public announcements on social media platforms, television interviews, referrals from physicians, and direct contact by the research team. The diversity of this recruitment strategy was intended to ensure a representative sample, thereby enhancing the generalizability of the study findings.

### Inclusion and exclusion criteria

Eligible participants were diagnosed with PD and fulfilled the following criteria: (a) adherence to the Queen Square Brain Bank (QSBB) diagnostic criteria (Hughes et al., 1992), excluding the “family history” component; and (b) a Hoehn & Yahr (H&Y) stage 1 or 2. Exclusion criteria included: (a) a clinical diagnosis of dementia based on consensus guidelines (Emre et al., 2007); (b) conditions meeting the MDS PD diagnosis exclusion criteria (Postuma et al., 2015); (c) linguistic barriers, specifically an inability to comprehend Brazilian Portuguese; (d) presence of advanced chronic organic diseases, as determined by the principal investigator; (e) claustrophobia; (f) pregnancy; and (g) contraindications to MRI, such as cardiac pacemakers, intracranial aneurysm clips, or electrodes for deep brain stimulation. The principal investigator made the final determination regarding exclusion due to dementia, integrating all available clinical information, measures of daily functioning, and MDS PDD criteria (Emre et al., 2007).

### Procedures

#### Clinical and cognitive assessment

Participants underwent evaluation during the ‘on’ phase of their levodopa therapy. A single neurologist assessed motor symptoms using Part III of the Movement Disorder Society-Unified Parkinson’s Disease Rating Scale (MDS-UPDRS) (Goetz et al., 2007) and the H&Y staging (Hoehn and Yahr, 1967). Demographic and clinical data, including years of education, age at disease onset, current medications, and dosages, were collected through interviews. Non-motor symptoms were evaluated using the Non-Motor Symptoms Scale (NMSS) (Chaudhuri et al., 2007). Levodopa equivalent daily dose (LEDD) was calculated according to established methods (Tomlinson et al., 2010). Participants were required to have stable dopaminergic medication dosages for at least 1 month prior to enrollment.

Global cognitive function was assessed using the PD-CRS (Pagonabarraga et al., 2008; Brandão et al., 2023) and MoCA (Nasreddine et al., 2005; Cesar et al., 2019). The PD-CRS is composed of nine tasks that generate “subcortical” and “cortical” subscores (Pagonabarraga et al., 2008). The subcortical subscore incorporates verbal fluency, immediate and delayed free-recall verbal memory, alternating verbal fluency, action verbal fluency, and clock drawing tasks. The cortical subscore includes confrontation naming and clock copying tasks. The Brazilian Portuguese PD-CRS transculturally-adapted version was validated by our group with large and diverse norms from more than 50 different Brazilian municipalities, adjusted for age and education attainment (Brandão et al., 2023).

The MDS-UPDRS Parts I, II, and IV were used to assess non-motor experiences of daily living, motor experiences of daily living, and motor complications, respectively (Goetz et al., 2007). Anxiety and depression were evaluated using the Hospital Anxiety and Depression Scale (HADS) (Faro and Faro, 2015). The REM Sleep Behavior Disorder Screening Questionnaire (RBDSQ) was employed to assess sleep disturbances (Pena-Pereira et al., 2020). Quality of life was measured using the Parkinson’s Disease Questionnaire-8 (PDQ-8) Summary Index (Luo et al., 2009). Depressive symptoms were further evaluated using the Beck Depression Inventory (BDI) (Beck et al., 1961; Gorenstein and Andrade, 1996).

All participants underwent a comprehensive neuropsychological assessment covering five cognitive domains. The evaluation covered attention and working memory [Digit Span Forward (DSF) (do Nascimento, 1993), Trail Making Test Part A (TMT-A) (Campanholo et al., 2014; Carvalho and Caramelli, 2020), Symbol Digit Modalities Test (SDMT) (Spedo et al., 2015; Pascoe et al., 2018)], executive functions [Trail Making Test Part B (TMT-B) (Campanholo et al., 2014; Carvalho and Caramelli, 2020), CANTAB One Touch Stockings of Cambridge (OTS) and Spatial Working Memory (SWM) (Robbins et al., 1994, 1998), Digit Span Backwards (do Nascimento, 1993) (DSB)], language [WAIS-III Similarities subtest (do Nascimento, 1993), PD-CRS Confrontation Naming (Brandão et al., 2023)], memory [CANTAB Paired Associates Learning [parênteses], Pattern Recognition Memory (PRM), and Verbal Recognition Memory (VRM) (Robbins et al., 1994, 1998)], and visuospatial function [PD-CRS Clock Drawing (Brandão et al., 2023)]. These tests have been chosen based on their robustness and widespread use in cognitive research, also ensuring our study’s findings are comparable to other landmark studies, such as ICICLE-PD (Yarnall et al., 2014; Mak et al., 2015; Firbank et al., 2017; Lawson et al., 2021). The incorporation of extensive electronic neurocognitive assessments from the CANTAB battery allowed for standardized testing (Robbins et al., 1994, 1998), ensuring consistency across assessments.

#### Cognitive grouping

The cognitive status classification approach adheres to a previously described method (Pereira et al., 2014), utilizing a modified approximation of the Movement Disorder Society Task Force Criteria (MDS TFC) (Litvan et al., 2012). This adaptation encompasses a battery of tests that collectively span four key cognitive domains – attention, memory, executive functions, and language – while exclusively featuring one task for the visuospatial domain. Minor adaptations to the MDS TFC protocol are elaborated upon subsequently.

The initial assessment focused on identifying subjective cognitive complaints. Such a complaint was considered present if the participant affirmed one or more items in Domain 5 of the Non-Motor Symptoms Scale (NMSS) (Carod-Artal and Martinez-Martin, 2013), specifically items 16, 17, and 18, which assess attentional acuity and memory related to facts, events, and tasks. Additionally, a score of at least one point on Item 1.1 of the Movement Disorder Society-Unified Parkinson’s Disease Rating Scale (MDS-UPDRS) (Goetz et al., 2007), which evaluates cognitive dysfunction, indicated subjective cognitive complaints.

To classify a case as PD-MCI, then, two primary conditions must have been met: (a) the presence of a subjective cognitive complaint; and (b) either impaired performance on a minimum of two tests within the same cognitive domain or impaired performance on one test in two or more cognitive domains. Impaired performance on each test was operationally defined as a score falling below-2.0 standard deviations compared to age-and education-adjusted local normative data. This criterion aligns with recommendations delineated in two previous studies (Goldman et al., 2013, 2015).

#### MRI acquisition

MRI data were acquired using a 3-Tesla Philips Intera Achieva scanner (Philips Medical Systems, Eindhoven, The Netherlands) with an 8-channel SENSE head coil. MRI protocols followed those delineated by the Alzheimer’s Disease Neuroimaging Initiative-3 (ADNI-3) (Weiner et al., 2017). Specifically, the T1-weighted images (T1WI) were acquired with the following parameters: a 3D-T1 Turbo Field Echo (TFE) sequence, a Field of View (FOV) measuring 208 × 240 × 256, a reconstructed spatial resolution of 1 × 1 × 1 mm, Echo Time (TE) set to minimum full echo, Repetition Time (TR) of 2,300 ms, and Inversion Time (TI) of 900 ms. The acquisition was accelerated by a factor of two.

#### Image processing

Cortical reconstruction and volumetric segmentation were performed using FreeSurfer software suite, version 7.1.1 (Fischl, 2012). The processing pipeline involved intensity normalization, skull stripping, Talairach transformation, subcortical segmentation, and triangular tessellation of the gray/white matter boundary (Reuter et al., 2010). It was followed by surface deformation to delineate both WM–GM and GM–pial boundaries. Subsequent steps included surface inflation, spherical atlas registration, and cortical parcellation of gyral and sulcal structures (Fischl et al., 1999). Cortical thickness measurements were derived as the distance between the GM-WM interface and its corresponding pial surface. The Desikan-Killiany atlas was used to parcellate the cerebral cortex into regions of interest (Desikan et al., 2006). To enhance data quality, smoothing was conducted using a 15 mm full-width-at-half-maximum (FWHM) symmetric Gaussian Kernel. For visual representation, significant cortical areas identified in the study were overlaid on a cortical surface model.

### Imaging quality control

Quality control was conducted to validate the fidelity of the processing steps and volume labeling. For each dataset, segmentations and parcellations were visually inspected. The FreeView tool facilitated the examination of T1WI sequences, identifying any segmentation inaccuracies or motion artifacts. This was accomplished through overlaying FreeSurfer output data onto raw MRI images.

### Data analysis

Demographic and clinical data were analyzed using R (version 4.1.0, R Foundation for Statistical Computing, Vienna, Austria). Continuous variables were compared using linear models. Categorical variables were compared using chi-squared tests.

Generalized linear models were employed to evaluate neuroimaging data, adjusted for confounding demographic and clinical variables such as sex, age and educational level. When conducting multiple simultaneous tests, *p*-values were adjusted using Benjamini and Hochberg’s false discovery rate (FDR) method (Benjamini and Hochberg, 1995).

The data predominantly had a continuous nature, thereby enhancing statistical power. Measures of central tendency and dispersion were the mean and standard deviation. Given the heterogeneous nature of the data distribution, regression models were preferred, which were applied across sociodemographic, clinical, and neuroimaging variables while duly controlling for confounders.

General linear models (GLM) were employed for vertex-wise analysis of CTh and CVol across each cerebral hemisphere, utilizing the ‘mri_glmfit’ function of the FreeSurfer software suite. The independent variables incorporated into the GLM included specific subtest scores from the PD-CRS, as well as aggregated scores categorizing ‘frontal-subcortical’ and ‘posterior cortical’ domains. To robustly address the issue of multiple comparisons, a permutation-based resampling approach was applied, involving 1,000 iterations. Significant clusters were identified using an FWE-corrected cluster-wise significance threshold of *p* < 0.05, adhering to current simulation-based guidelines designed to minimize the likelihood of false-positive results (Greve and Fischl, 2018). Spatial localization of the brain regions of interest was conducted using the Montreal Neurological Institute (MNI) coordinate system (Fischl, 2012).

To examine differences in CTh between PD-MCI and PD-IC groups, we employed a GLM for vertex-wise analysis. In this model, CTh served as the dependent variable, while potential confounding factors, including age, sex, and educational level, were controlled for as covariates. A separate GLM was constructed to evaluate CVol as the dependent variable. This model incorporated the same confounding factors as the CTh analysis, with the addition of total intracranial volume as a nuisance variable to account for individual differences in overall brain size. This comprehensive approach allowed us to isolate the effects of cognitive status on cortical structure while minimizing the influence of demographic and physiological variables known to impact brain morphometry.

## Results

### Participants

**Figure 1:**
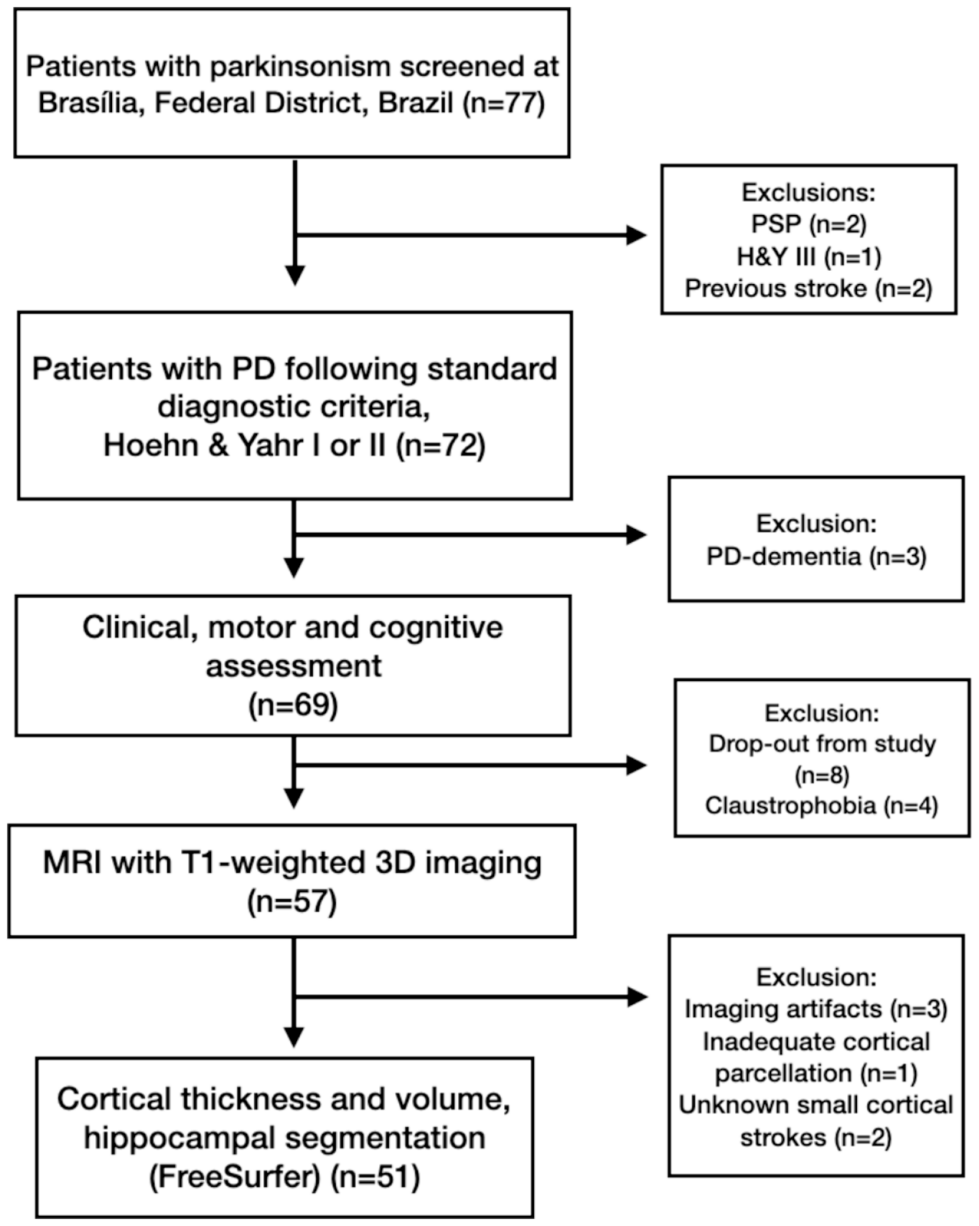
Participant flowchart for the MRI study: attrition and exclusion metrics.

Our study initially screened a cohort of 77 individuals presenting with Parkinsonism between March 2019 and August 2021, as shown in the study flowchart (Figure 1). Of these, three patients with PD were subsequently excluded due to a comorbid diagnosis of dementia. Additionally, two individuals were diagnosed with atypical parkinsonism: one with Progressive Supranuclear Palsy of the Parkinsonian Subtype (PSP-Parkinsonism) and the other with PSP-Richardson syndrome. Finally, another three PD patients were excluded, two due to a history of cerebrovascular events, and one PD who was restaged as Hoehn and Yahr stage III. Consequently, an initial group of 69 PD patients was invited to partake in the MRI component of the study.

Post-enrollment drop-out occurred due to various factors, including claustrophobia, concerns regarding SARS-CoV-2 infection amid the COVID-19 pandemic, and MRI acquisitions that failed to meet the requisite quality control standards (e.g., due to motion artifacts or inadequate segmentation by the FreeSurfer software). Comprehensive demographic and clinical characteristics of the remaining sample after these dropouts are delineated in Table 1. The final sample designated for CTh and CVol evaluation consisted of 51 PD patients, divided into PD-MCI (*n* = 14, 27.4%) and PD-IC (*n* = 37, 72.5%), as described in Figure 1.

**Table 1:** Clinical and demographic characteristics of PD-IC and PD-MCI patients in the Brasilia cohort.

Clinical or demographic variable	PD-IC (*n* = 37)	PD-MCI (*n* = 14)	*p*
Sex			0.791
Female, *n* (%)	12 (32.4%)	4 (28.6%)	
Male, *n* (%)	25 (67.6%)	10 (71.4%)	
Age (years)	59.0 ± 11.0	63.4 ± 9.5	0.198
Educational level (years)	15.6 ± 4.0	12.6 ± 4.1	0.022*
Disease duration (years)	4.2 (3.3)	4.1 (3.1)	0.869
Hoehn & Yahr			0.323
Stage 1	19 (51.4%)	5 (35.7%)	
Stage 2	18 (48.6%)	9 (64.3%)	
MDS-UPDRS part 1	9.4 ± 6.2	14.6 ± 9.2	0.024*
MDS-UPDRS part 2	8.3 ± 7.2	12.6 ± 9.0	0.081
MDS-UPDRS part 3	22.4 ± 11.3	26.3 ± 13.7	0.303
MDS-UPDRS part 4	3.4 ± 4.5	3.1 ± 3.2	0.826
HADS-anxiety	5.6 ± 3.3	6.6 ± 3.8	0.338
HADS-depression	5.2 ± 3.1	6.8 ± 5.0	0.189
MoCA	26.08 ± 2.12	22.2 ± 3.61	<0.001*
F letter fluency	14.1 ± 4.7	11.1 ± 3.3	0.036*
RBDSQ	5.6 ± 3.0	6.9 ± 2.7	0.165
PDQ-8 SI	24.9 ± 14.2	37.9 ± 21.0	0.014*
BDI	9.0 ± 5.5	11.7 ± 8.0	0.170
NMSS	41.5 ± 30.0	73.3 ± 53.2	0.009*
1. Cardiovascular/falls	1.5 ± 3.4	2.5 ± 2.8	0.345
2. Sleep/fatigue	6.9 ± 7.1	13.8 ± 13.3	0.020*
3. Mood/apathy	7.8 ± 10.3	17.8 ± 18.3	0.017*
4. Perceptual/hallucinations	0.9 ± 2.0	2.6 ± 4.9	0.079
5. Attention/memory	4.6 ± 7.0	12.3 ± 12.2	0.007*
6. Gastrointestinal	3.7 ± 4.7	6.3 ± 8.1	0.164
7. Urinary	6.1 ± 8.6	5.9 ± 7.6	0.916
8. Sexual	5.1 ± 4.8	7.1 ± 5.2	0.206
9. Miscellaneous	4.8 ± 4.4	5.1 ± 5.2	0.821

The clinical and demographic data of patients with Parkinson’s Disease with intact cognition (PD-IC) and those with mild cognitive impairment (PD-MCI) were analyzed using linear regression for continuous, chi-squared for categorical and Trend test for ordinal variables. (*) indicates statistically significant values. MDS-UPDRS, Movement Disorder Society-Unified Parkinson’s Disease Rating Scale; HADS, Hospital Anxiety and Depression Scale; MoCA, Montreal Cognitive Assessment; RBDSQ, REM Sleep Behavior Disorder Screening Questionnaire; PDQ-8, Parkinson’s Disease Questionnaire-8; SI, Summary Index; BDI, Beck Depression Inventory; NMSS, Non-Motor Symptoms Scale.

Table 1 presents a comparison of clinical and demographic characteristics between PD-IC (*n* = 37) and those with PD-MCI (*n* = 14) from the cohort. Significant differences (*p* < 0.05) were observed in several parameters, including educational level, MDS-UPDRS part 1, MoCA scores, F letter fluency, PDQ-8 SI, and NMSS total score. Notably, PD-MCI patients exhibited lower educational levels (12.6 ± 4.1 vs. 15.6 ± 4.0 years, *p* = 0.022), higher MDS-UPDRS part 1 scores (14.6 ± 9.2 vs. 9.4 ± 6.2, *p* = 0.024), and lower MoCA scores (22.2 ± 3.61 vs. 26.08 ± 2.12, *p* < 0.001) compared to PD-IC patients. Additionally, PD-MCI patients demonstrated significantly higher NMSS total scores (73.3 ± 53.2 vs. 41.5 ± 30.0, *p* = 0.009), with specific domains such as sleep/fatigue, mood/apathy, and attention/memory showing marked differences. These findings suggest that PD-MCI patients experienced more severe non-motor symptoms compared to their PD-IC counterparts.

### Cognitive profiles

Table 2 reveals relevant cognitive disparities between individuals with PD-IC and those with PD-MCI across several cognitive domains. In the attention domain, the Symbol Digit Modalities Test (SDMT) demonstrated significantly lower scores in the PD-MCI group (*p* < 0.001 for both *Z*-scores and raw scores), suggesting substantial attention deficits. While no significant differences were found in the Digit Span Forward (DSF) test, the Trail Making Test Part A (TMT-A) results indicated impaired processing speed and sustained attention in PD-MCI (*p* < 0.001 for both *Z*-scores and raw scores).

**Table 2:** Cognitive profiles of participants with PD-MCI and PD-IC divided into cognitive domains according to the MDS TFC.

Cognitive domain	Cognitive test	PD-IC (*n* = 37)		PD-MCI (*n* = 14)		*p*-value	
		*Z*-scores (mean ± SD, range)	Raw scores (mean ± SD, range)	*Z*-scores (mean ± SD, range)	Raw scores (mean ± SD, range)	*Z*-scores	Raw scores
Attention	SDMT	0.1 ± 0.6 (−1.4 to 1.1)	42.9 ± 9.4 (23.0–60.0)	−0.7 ± 1.0 (−3.2 to 1.2)	28.3 ± 14.3 (4.0–52.0)	0.001*	<0.001*
	DSF	0.3 ± 1.2 (−1.5 to 3.3)	9.8 ± 2.6 (5.0–16.0)	0.0 ± 1.2 (−1.7 to 1.7)	8.3 ± 2.7 (5.0–12.0)	0.356	0.073
	TMT-A	0.3 ± 0.7 (−2.6 to 1.4)	44.2 ± 17.0 (18.0–118.0)	−1.3 ± 2.0 (−4.8 to 1.0)	87.1 ± 42.8 (34.0–150.0)	<0.001*	<0.001*
Memory	PRM (delayed recognition)	0.0 ± 1.0 (−2.6 to 1.4)	84.5 ± 13.6 (50.0–100.0)	−0.8 ± 1.3 (−4.0 to 0.8)	71.4 ± 16.9 (33.3–91.7)	0.013*	0.006*
	VRM (delayed recognition)	−0.2 ± 0.9 (−2.5 to 1.7)	30.1 ± 2.8 (23.0–36.0)	−0.9 ± 1.5 (−2.8 to 1.3)	28.5 ± 3.7 (22.0–35.0)	0.047*	0.115
	PAL (total errors adjusted)	−0.4 ± 0.9 (−2.4 to 0.6)	21.4 ± 14.9 (3.0–57.0)	−0.7 ± 1.0 (−2.7 to 0.6)	30.1 ± 19.0 (4.0–64.0)	0.232	0.094
Executive functions	OTS (problems solved 1st try)	−0.4 ± 1.2 (−2.9 to 1.4)	8.7 ± 3.7 (1.0–15.0)	−1.5 ± 0.8 (−3.1 to 0.2)	5.6 ± 3.1 (0.0–11.0)	0.004*	0.007*
	SWM (between-errors)	0.1 ± 1.1 (−1.1 to 1.8)	18.5 ± 7.6 (0.0–31.0)	−0.4 ± 0.9 (−1.1 to 1.6)	21.2 ± 6.8 (7.0–31.0)	0.189	0.239
	TMT (B-A)	0.3 ± 0.8 (−2.5 to 1.6)	48.6 ± 36.0 (12.0–182.0)	−1.3 ± 1.5 (−4.0 to 0.8)	119.4 ± 59.8 (35.0–242.0)	<0.001*	<0.001*
	DSB	−0.4 ± 0.8 (−1.5 to 3.3)	8.3 ± 2.6 (5.0–16.0)	−0.7 ± 0.9 (−1.7 to 1.7)	7.4 ± 2.8 (5.0–12.0)	0.239	0.094
Visuospatial	Clock copying (PD-CRS)	0.2 ± 0.6 (−2.6 to 1.4)	9.8 ± 0.5 (8.0–10.0)	−0.8 ± 2.5 (−4.0 to 0.8)	8.9 ± 2.0 (4.0–10.0)	0.029*	0.007*
Language	Figure naming (PD-CRS)	0.6 ± 0.6 (−0.7 to 1.4)	18.4 ± 1.6 (15.0–20.0)	0.7 ± 0.5 (−0.1 to 1.4)	17.9 ± 1.6 (15.0–20.0)	0.589	0.294
	Similarities (WAIS-III)	1.4 ± 0.6 (−0.2 to 2.3)	30.5 ± 5.0 (16.0–38.0)	0.8 ± 0.7 (−0.3 to 1.8)	25.6 ± 5.6 (15.0–33.0)	0.002*	0.003*

DSB, Digit Span Backward; DSF, Digit Span Forward; MDS TFC, Movement Disorder Society Task Force Criteria; OTS, One-Touch Stockings of Cambridge; PAL, Paired Associates Learning; PD-CRS, Parkinson’s Disease-Cognitive Rating Scale; PD-IC, Parkinson’s Disease with Intact Cognition; PD-MCI, Parkinson’s Disease with Mild Cognitive Impairment; PRM, Pattern Recognition Memory; SDMT, Symbol Digit Modalities Test; SWM, Spatial Working Memory; TMT-A, Trail Making Test Part A; TMT(B-A), Subtraction TMT-A from TMT-Part B; VRM, Verbal Recognition Memory; WAIS-III, WAIS-III Similarities Subtest; *p < 0.05. The *Z*-scores for performance in each neuropsychological instrument are based on a local control group, adjusting for age and education level. The statistical analyses were performed using a linear model ANOVA.

Memory domain tests showed mixed results. Pattern Recognition Memory (PRM) and Verbal Recognition Memory (VRM) delayed recognition tasks revealed worse performance in PD-MCI individuals (PRM: *p* = 0.013 for *Z*-scores, *p* = 0.006 for raw scores; VRM: *p* = 0.047 for *Z*-scores), particularly in delayed recognition. However, associative learning measured by the Paired Associates Learning (PAL) test did not differ significantly between groups.

Executive function assessments revealed notable deficits in PD-MCI participants. The One Touch Stockings of Cambridge test, an electronic version of the Tower of London, showed impaired problem-solving abilities in the PD-MCI group (*p* = 0.004 for *Z*-scores, *p* = 0.007 for raw scores). The TMT (B-A) test highlighted impaired cognitive flexibility in PD-MCI (*p* < 0.001 for both *Z*-scores and raw scores). However, spatial working memory, as measured by the SWM between-errors test, and working memory, assessed by Digit Span Backward (DSB), appeared relatively unaffected.

Visuospatial abilities, assessed through clock copying from the PD-CRS, were significantly poorer in the PD-MCI group (*p* = 0.029 for *Z*-scores, *p* = 0.007 for raw scores). Language abilities showed mixed results: naming abilities, measured by the PD-CRS Figure Naming test, were preserved, but abstraction, as measured by the WAIS-III similarities test, was impaired in the PD-MCI group (*p* = 0.002 for *Z*-scores, *p* = 0.003 for raw scores).

Table 3 evidences a significant disparity in several PD-CRS subtests between patients classified as PD-MCI and PD-IC. Specifically, the Immediate Memory and Clock Copying tests show significant cognitive declines in the PD-MCI group (*p* = 0.004 and *p* = 0.007, respectively), a pattern echoed in Clock Drawing and Action Verbal Fluency (*p* = 0.019 and *p* = 0.016). The most relevant differences are noted in Alternating Fluency and the overall PD-CRS score (*p* < 0.001). In contrast, tests like Naming, Sustained Attention, and Delayed Memory Recall did not exhibit notable differences, suggesting some cognitive functions remained relatively intact in the PD-MCI group.

**Table 3:** Comparison of PD-CRS subtest scores between PD-MCI and PD-IC groups.

PD-CRS subtest	PD-MCI, mean (SD)	PD-IC, mean (SD)	Total PD sample, mean (SD)	*p*-value
Immediate memory	8.6 (1.7)	10.2 (1.6)	9.7 (1.8)	0.004*
Naming	17.9 (1.6)	18.4 (1.6)	18.2 (1.6)	0.294
Sustained attention	8.6 (1.5)	9.2 (1.5)	9.1 (1.5)	0.223
Working memory	5.9 (2.3)	7.2 (2.0)	6.8 (2.2)	0.063
Clock drawing	7.8 (2.8)	9.2 (1.3)	8.8 (1.9)	0.019*
Clock copying	8.9 (2.0)	9.8 (0.5)	9.5 (1.2)	0.007*
Delayed memory recall	5.2 (2.5)	6.4 (2.9)	6.1 (2.8)	0.183
Alternating fluency	7.4 (3.1)	11.7 (4.0)	10.5 (4.2)	<0.001*
Action verbal fluency	13.9 (3.0)	18.1 (6.1)	16.9 (5.7)	0.016*
Frontal-subcortical score	72.0 (13.0)	57.4 (9.9)	68.0 (13.8)	<0.001
Posterior cortical score	28.2 (1.8)	26.7 (2.4)	27.8 (2.1)	0.021
PD-CRS total	84.1 (11.3)	100.2 (13.5)	95.8 (14.7)	<0.001*

PD, Parkinson’s disease; PD-MCI, Parkinson’s disease with mild cognitive impairment; PD-IC, Parkinson’s disease with intact cognition; SD, standard deviation; PD-CRS, Parkinson’s disease, Cognitive Rating Scale; *Statistical significant group difference in the linear model ANOVA.

### Cortical volume and thickness

#### PD-MCI versus PD-IC

**Figure 2:**
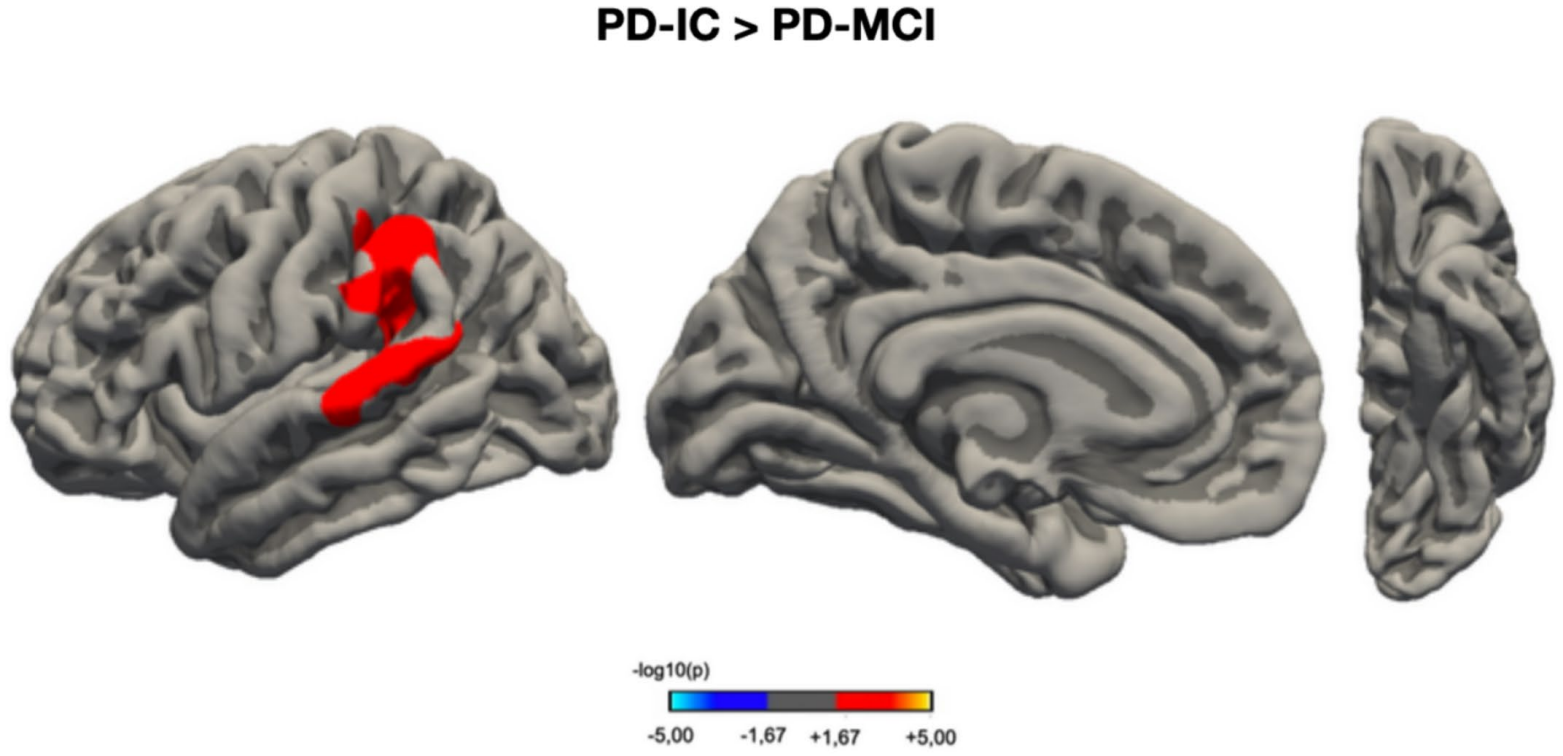
Vertex-wise analysis of cortical volume differences between PD-IC and PD-MCI groups. This figure presents a statistical parametric map illustrating regions of significant cortical volume (CVol) differences between Parkinson’s disease patients with intact cognition (PD-IC, *n* = 37) and those with mild cognitive impairment (PD-MCI, *n* = 14). The results are projected onto a standardized cortical surface template. The color scale represents the statistical significance of group differences, expressed as −log10(*p*-value), where warmer colors indicate greater statistical significance. This visualization allows for the spatial localization of cortical regions most affected by cognitive status in Parkinson’s disease. PD-IC, Parkinson’s disease with intact cognition; PD-MCI, Parkinson’s disease with mild cognitive impairment; CVol, cortical volume.

The group factor’s impact (PD-MCI vs. PD-IC) on CVol was analyzed through distinct GLMs for each cerebral hemisphere. The observed CVol disparities between the PD-IC and PD-MCI cohorts were presented in a cluster encompassing 2,394 mm^2^ surface area of the left hemisphere (*p*_FWER_ = 0.01594). The coordinates in the Montreal Neurological Institute (MNI) space (Evans et al., 1993) for this cluster were *x* = −57.1, *y* = −38.0, *z* = 39.5. This region predominantly comprises the superior temporal, supramarginal, and superior parietal cortices, also marginally including the superior temporal sulcus and a minor section of the postcentral gyrus. This cluster is visually delineated in Figure 2. The results are presented in the format of −log10(*p*) maps, where a statistical significance threshold of *p* < 0.05 corresponds to −log10(*p*) values exceeding +1.3. Notably, no statistically significant variances in CTh were identified between the PD-MCI and PD-IC groups.

#### Correlations between PD-CRS scores and cortical architecture in non-demented PD patients

Within our combined sample of non-demented PD patients, comprising both PD-IC and PD-MCI who were at Hoehn and Yahr stages 1–2 (HY 1–2), we observed noteworthy associations between the scores on the PD-CRS and variations in CTh. These correlations were statistically controlled for demographic confounders such as age and sex. The subsequent delineation of these findings is described below.

#### Associations between PD-CRS total and subtest scores and cortical volumes

**Figure 3:**
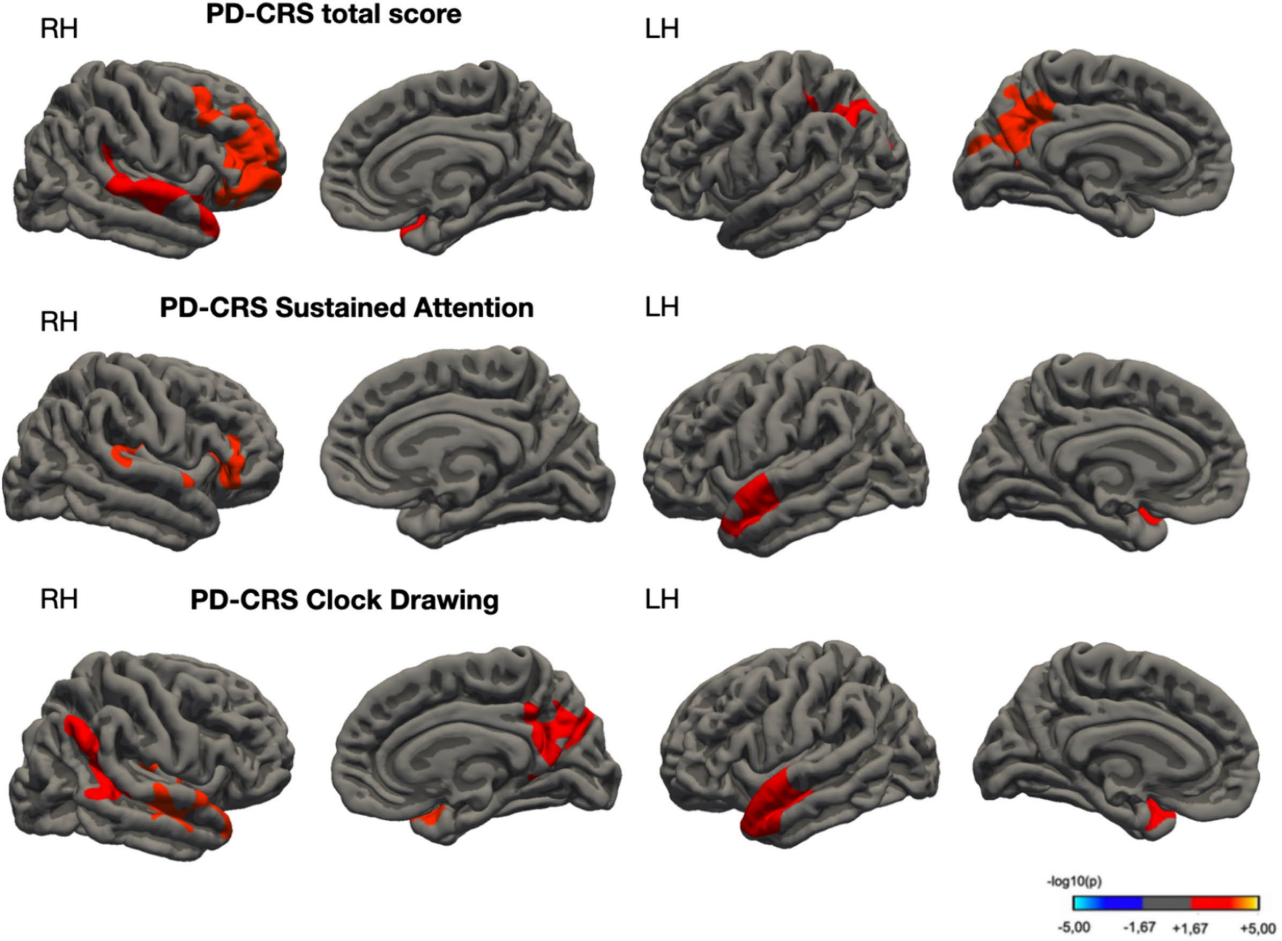
Cortical regions exhibiting significant associations with PD-CRS scores across multiple domains in Parkinson’s disease cohort. Subpanels: The top subpanel illustrates areas correlated with the PD-CRS total score, the middle with Sustained Attention, and the bottom with Clock Drawing Test scores. Statistical significance was determined using permutation-based testing with 1,000 iterations at a threshold of *p* < 0.05. The color scale represents the strength of the correlation, expressed as −log10(*p*-value), where warmer colors indicate stronger associations.

Figure 3 provides a graphical representation of the relationship between the PD-CRS total scores and cortical volumes, indicating associations with two distinct vertex clusters in each hemisphere. These associations are enumerated in Table 4. Similarly, the sustained attention subtest demonstrated a significant relationship with a single vertex cluster in each hemisphere. For the Clock Drawing Test, three significant vertex clusters were identified in the right hemisphere and one in the left, all of which are elaborated upon in Figure 3 and Table 4.

**Table 4:** Spatial coordinates and the extent of areas showing a significant association between cortical thickness and PD-CRS cognitive tests (total score, sustained attention, and clock drawing).

MNI coordinates			Side	Anatomical location	Cluster size, mm^2^	*p*_FWER_
*x*	*y*	*z*				
PD-CRS total score						
25.7	48.5	8.9	R	Rostral middle frontal	5,478	0.0002
42.2	16.1	−28.7	R	Superior temporal	2,546	0.0008
−12.7	−49.0	29.5	L	Isthmus cingulate	2,982	0.0002
−37.3	−64.8	42.6	L	Inferior parietal	2,266	0.0016
PD-CRS sustained attention						
38.4	−26.6	5.0	R	Insula	2,913	0.0002
−42.7	−3.1	−21.4	L	Superior temporal	1,787	0.01236
PD-CRS clock drawing						
35.0	−18.8	11.3	R	Insula	2,834	0.0002
18.4	−74.0	36.8	R	Superior parietal	2,280	0.0034
45.0	−45.7	16.3	R	Inferior parietal	1,765	0.01732
−53.1	−0.8	−7.7	L	Superior temporal	2,357	0.0012

MNI, Montreal Neurological Institute, R, right hemisphere; L, left hemisphere; PD-CRS, Parkinson’s Disease-Cognitive Rating Scale; *p*_FWER_, *p*-values corrected for family-wise error rate. The coordinates *x*, *y*, and *z* refer to the neuroanatomical location in the standard stereotactic space. Anatomical locations represent the vertices with the lowest *p*-value. Age and educational level were nuisance variables in the general linear model.

**Figure 4:**
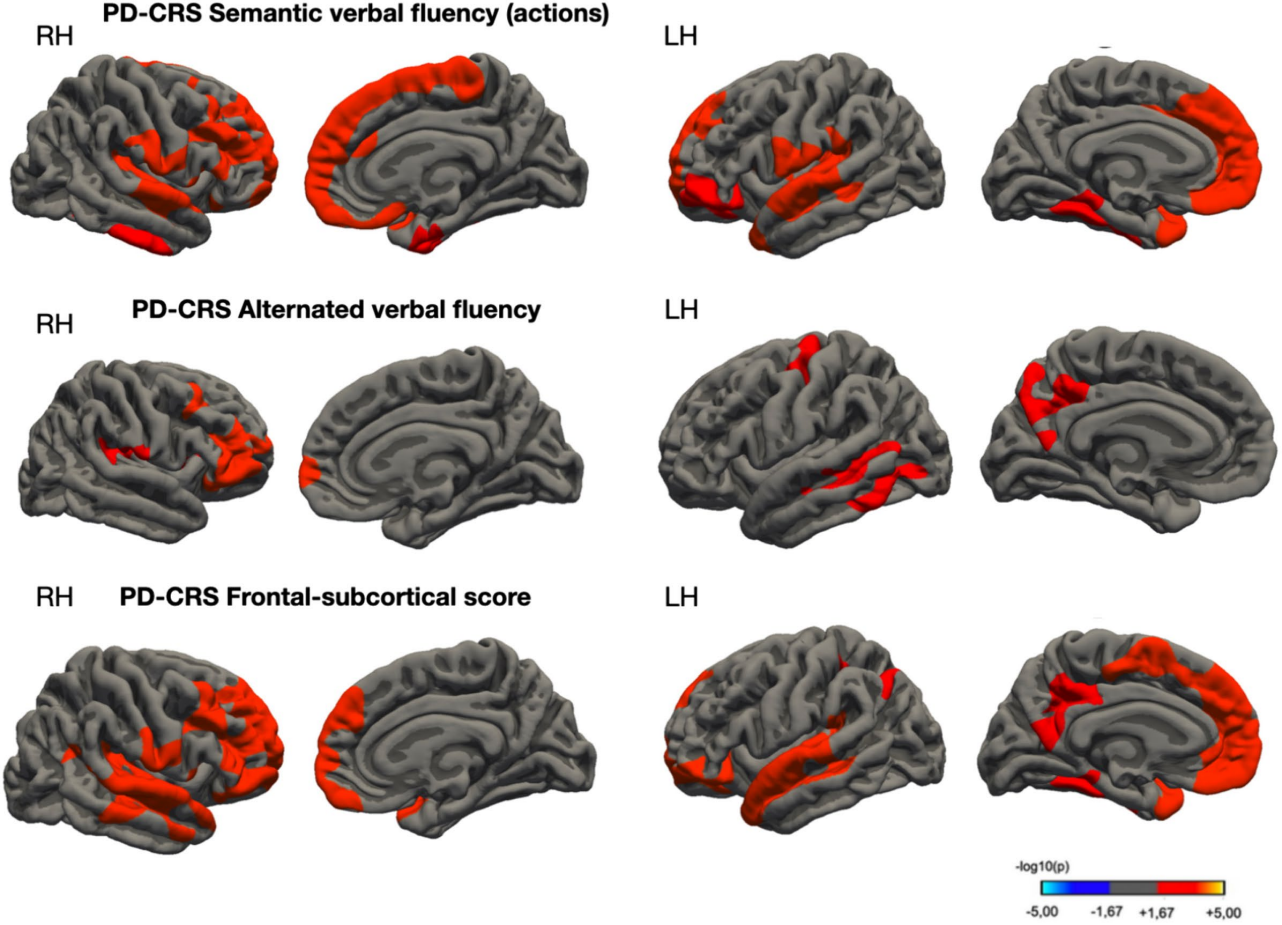
Cortical thickness areas showed significant correlations with PD-CRS semantic verbal fluency (upper panel), alternated verbal fluency (between letter S and clothing items, in the middle panel), and Frontal-Subcortical Score (lower panel) in a sample of non-demented PD patients. Color scale indicates correlation strength [−log10(*p*-value)]. Significance determined by permutation testing (1,000 iterations, *p* < 0.05).

Table 5 and Figure 4 delineate the structural brain correlates associated with semantic and alternating verbal fluencies and the Frontal-Subcortical score. Notably, no significant cortical clusters were observed concerning other subtests of the PD-CRS, including immediate and delayed memory recall, figure naming, clock copy, and posterior cortical score.

**Table 5:** Spatial coordinates and extent of areas showing a significant association between cortical thickness (CTh) and other PD-CRS cognitive tests (semantic verbal fluency, alternated verbal fluency, and frontal-subcortical score).

MNI coordinates			Side	Anatomical location	Cluster size, mm^2^	*p*_FWER_
*x*	*y*	*z*				
PD-CRS semantic verbal fluency (actions)						
31.0	39.1	−8.1	R	Lateral orbitofrontal	3,219	0.0002
43.3	−22.9	4.1	R	Transverse temporal	1,686	0.0002
−7.8	43.7	−13.7	L	Medial orbitofrontal	3,038	0.0002
−57.4	−5.7	−1.6	L	Superior temporal	1,117	0.002
−33.8	−45.7	−7.8	L	Fusiform	773	0.02326
PD-CRS alternating verbal fluency						
41.0	39.2	−3.2	R	Pars triangularis	4,038	0.0002
37.5	−13.7	21.3	R	Insula	1,869	0.01296
−53.1	−60.0	−8.3	L	Inferior temporal	2,340	0.0012
−32.3	−20.3	46.3	L	Precentral	1,712	0.02049
−10.0	−40.4	38.4	L	Precuneus	1,702	0.02148
PD-CRS frontal-subcortical score						
40.1	−25.8	4.5	R	Transverse temporal	15,979	0.0002
−7.6	44.1	−14.2	L	Medial orbitofrontal	5,847	0.0002
−57.4	−3.6	−3.0	L	Superior temporal	4,825	0.0002
6.5	−38.9	40.5	L	Precuneus	1,950	0.00659
−34.2	−44.5	−8.3	L	Fusiform	1,850	0.00958
−33.9	−41.4	39.5	L	Supramarginal	1,633	0.03017

MNI, Montreal Neurological Institute, R, right hemisphere; L, left hemisphere; PD-CRS, Parkinson’s Disease-Cognitive Rating Scale; *p*_FWER_, *p*-values corrected for family-wise error rate. The coordinates *x*, *y*, and *z* refer to the neuroanatomical location in the standard stereotactic space. According to DK atlas boundaries, anatomical locations represent the vertices with the lowest *p*-value. Age and educational level were nuisance variables in the general linear model.

## Discussion

This study provides a comprehensive analysis of cognitive and cerebral structural differences between PD-IC and those with PD-MCI. Our findings reveal significant distinctions between these groups across multiple domains, including clinical characteristics, cognitive profiles, and neuroanatomical correlates. In terms of clinical and demographic characteristics, the PD-MCI group presented a distinct profile, characterized by lower educational attainment and higher prevalence of non-motor symptoms (evidenced by higher scores on MDS-UPDRS part 1 and NMSS). These findings corroborate previous studies suggesting an association between lower cognitive reserve and increased risk of cognitive impairment in PD (Hindle et al., 2014, 2016). Tests such as SDMT, TMT-A, PRM, VRM, OTS, and TMT (B-A) demonstrated marked differences between the groups. These results align with the concept that PD-MCI is heterogeneous and affects multiple cognitive domains (Aarsland et al., 2010, 2021).

A particularly interesting finding of this study was the identification of significant differences in cortical volume between PD-MCI and PD-IC groups, primarily in left hemisphere regions, including superior temporal, supramarginal, and superior parietal cortices. These areas are associated with cognitive functions that frequently show decline in PD-MCI, such as working memory, attention, and visuospatial processing (Hanganu et al., 2014). Interestingly, while volumetric differences were observed, no significant disparities in CTh were detected between the groups. This discrepancy may be attributed to several factors. Firstly, our study’s relatively small sample size may have limited its statistical power to detect subtle CTh differences, potentially explaining inconsistencies with previous research (Pereira et al., 2014; Mak et al., 2015). Alternatively, this finding could suggest a temporal progression in structural brain changes associated with cognitive decline in PD, wherein volumetric alterations could precede detectable changes in cortical thickness. This hypothesis aligns with the concepts that these two measures (CTh and CVol) may provide complementary information in PD neurodegeneration research (Hutton et al., 2009; Pereira et al., 2012; Weintraub et al., 2012; Mak et al., 2014; Xu et al., 2016; Filippi et al., 2020; Li et al., 2022) and warrants further investigation through longitudinal studies with larger cohorts.

The observed correlations between PD-CRS scores and cortical volume in various brain regions provide additional evidence of the relationship between brain structure and cognitive function in PD. These associations were particularly notable for the PD-CRS total score and specific subtests such as sustained attention and clock drawing, suggesting that structural alterations in specific brain regions may underlie the cognitive deficits observed in PD-MCI. Lower total PD-CRS scores correlated with reduced CTh in regions including the middle frontal gyrus, superior temporal gyrus, inferior parietal lobule, and cingulate cortex. Moreover, scores related to frontal-subcortical domains showed positive correlations with CTh in various regions, including the transverse temporal gyrus, medial orbitofrontal cortex, superior temporal gyrus, insula, precuneus, fusiform gyrus, and supramarginal gyrus.

Our findings support the notion that semantic and alternating verbal fluency tasks are mediated by distinct neural circuits. Alternating verbal fluency tasks appear to rely on the lateral frontal lobe, precuneus, and posterior temporal regions, while semantic verbal fluency tasks are more closely associated with the dorsolateral prefrontal cortex (Biesbroek et al., 2016; Schmidt et al., 2017; Ghanavati et al., 2019). This dichotomy underscores the complexity of neural underpinnings in verbal fluency tasks, aligning with previous literature. For instance, Pereira et al. (2009) in a study with 32 PD patients, identified a correlation between reduced semantic fluency and gray matter loss in frontal and temporal regions, findings that resonate with our research. However, this study did not find a similar anatomical correlation for phonemic verbal fluency. Additionally, Segura et al. (2014) observed cortical thinning in areas associated with verbal fluency in PD-MCI patients compared to those without MCI (*n* = 47 and *n* = 43, respectively) (Segura et al., 2014). Their study also distinguished between cortical regions involved in phonemic versus semantic fluency. Using covariance analyses, they established connections between semantic fluency and temporal and parietal lobe regions. Our study partially corroborates these observations, noting significant links between temporal region cortical thickness and PD-CRS semantic fluency scores.

PD-CRS has emerged as a reliable diagnostic tool for discerning both executive (frontal) and posterior cortical deficits, hallmarks of the cognitive profile in PD-MCI (Pagonabarraga et al., 2008; Skorvanek et al., 2018; Brandão et al., 2023). These observations align with the dual syndrome hypothesis, as previous literature corroborates, including studies from a Spanish cohort (Carvalho and Caramelli, 2020). In patients classified as PD-MCI according to PD-CRS thresholds, notable cortical thinning is observed in regions such as the precuneus, supramarginal gyrus, and angular gyrus (Garcia-Diaz et al., 2018; Aracil-Bolaños et al., 2022). Our data further highlight the role of temporal and parietal cortical thinning in attentional, mnestic, and visuoperceptive tasks, consistent with prior studies (Pagonabarraga et al., 2013; Segura et al., 2014).

This study presents an intriguing contrast when juxtaposed with previous investigations into the neural correlates of categorical and alternating verbal fluency in PD-MCI patients. Earlier studies have suggested that these measures of fluency are associated with cortical thinning in areas including the cuneus, banks of the superior temporal sulcus, fusiform gyrus, and lingual gyrus among this patient population (Pagonabarraga et al., 2013). However, our analysis paints a more nuanced picture of these associations. While our findings align with some previously identified anatomical correlates, such as the precuneus and inferior temporal lobe, they also diverge in several key aspects. Notably, our study reveals a distinctive structural correlation between alternating verbal fluency and the anatomical integrity of the frontal lobe. This includes specific areas such as the *pars triangularis* of the inferior frontal gyrus, the insula, and precentral regions. These findings underscore the complexity of neural mechanisms underlying verbal fluency tasks in PD-MCI and highlight the need for continued exploration in this domain.

Regarding visuospatial function, earlier studies on Alzheimer’s disease have pinpointed the superior parietal cortex, precuneus, inferior temporal gyrus, and lateral occipital regions as key neural correlates of the Clock Drawing test (CDT) (Kang et al., 2019). Interestingly, our observations in a PD cohort largely echo these findings. Specifically, we have demonstrated a significant association between performance in the CDT and gray matter integrity in the parietal lobe, precuneus, superior temporal gyrus, and insula. These data suggest that a detailed evaluation of subtest performance on the PD-CRS could act as a predictive model for cerebral atrophy patterns in PD patients. In essence, these comparative analyses not only underscore the effectiveness of PD-CRS in capturing the complex cognitive deficits in PD-MCI but also provide refined insights into the structural neural correlates underlying diverse cognitive functions.

As mentioned, the CDT and its modified versions have been widely used to screen for cognitive impairment across various diseases, including PD. These tests critically assess important brain functions, including comprehension, motor programming and execution, visual memory, concentration, abstraction, and attention (Riedel et al., 2013; Hazan et al., 2018; Lolekha et al., 2021). However, studies focusing on the neural underpinnings of the CDT in PD are scarce. A recent study revealed that patients with early-stage PD exhibit slower activation of the right prefrontal cortex during clock drawing tasks, as determined by functional near-infrared spectroscopy (Schejter-Margalit et al., 2022). Garcia-Diaz et al. (2018) investigated brain structures linked to visuospatial and visuoperceptual tests in PD-MCI patients using a clock copying task (CLOX2) (Garcia-Diaz et al., 2018). Although CLOX2 performances were similar across the study groups, a correlation was found between test performance and cortical thickness in the precuneus and isthmus of the cingulate gyrus in PD patients. Another study linked precuneus thinning to clock copying, alongside the parahippocampal and lingual gyrus, predominantly in PD patients with dementia rather than MCI (Pagonabarraga et al., 2013). More recently, research has shown consistent atrophy patterns in patients with mild to moderate PD (Hoehn & Yahr Previously II and III), involving the insula, parahippocampal gyrus, medial temporal lobes, and precuneus. This indicates a progressive degeneration of these brain substrates as the disease advances (Benninger et al., 2023).

Our study agreed with some of the aforementioned brain substrates, as we observed a correlation between thinning in the insula and performance on the CDT. Additionally, our data revealed a connection between the integrity of the cingulate gyrus and the total score on the PD-CRS, as well as an association between the precuneus and both the PD-CRS alternating verbal fluency and frontal-subcortical scores. Aligning with our findings, recent research has underscored the role of the insula – regarded as a central neural hub – in cognitive dysfunction in PD. This study particularly highlighted the loss of connectivity between the insula and the anterior cingulate cortex as being critically relevant to cognitive impairment in PD patients (Fathy et al., 2020).

Building on these insights, the role of the precuneus emerges as crucial in understanding PD-related neurodegeneration (Thibes et al., 2017; Uribe et al., 2018; Jia et al., 2019). As delineated by Cavanna and Trimble (2006), the precuneus is a key player in visuospatial processing, the mental manipulation of spatial relationships, and the formation of spatial mental imagery – functions that are notably compromised in PD (Cavanna and Trimble, 2006; Garcia-Diaz et al., 2018). Its involvement extends to vital cognitive domains such as episodic memory, self-related cognition, consciousness, awareness, and executive functions, all of which are frequently affected in PD (Aarsland et al., 2017, 2021). The precuneus also has a significant role in integrating sensory information with internal cognitive processes (Cavanna and Trimble, 2006). This integration capacity is crucial, as it forms a bridge between external stimuli and internal experiences, a function that may be particularly disrupted in PD. Hence, the precuneus’s functions not only resonate with our findings but also provide a broader context for understanding the cognitive challenges faced by individuals with PD.

### Study strengths and limitations

This study has several strengths, including the use of comprehensive neuropsychological assessments adhering to Level II MDS Task Force criteria for PD-MCI classification, and the employment of advanced neuroimaging techniques to explore brain morphological correlates of cognitive performance. However, some limitations should be acknowledged.

Firstly, the classification of PD-MCI using Level II criteria resulted in a mean PD-CRS score that was slightly above the commonly cited cutoff for Level I screening (PD-CRS < 82) (Fernández de Bobadilla et al., 2013; Brandão et al., 2023). This discrepancy highlights the potential differences between Level I and II assessments, and underscores the complexity of MCI diagnosis in PD, which must account for educational and cultural factors specific to distinct populations (Brandão et al., 2020, 2023).

Secondly, the mean *Z* scores for neuropsychological tests in the PD-MCI group were not consistently below-1.5 SD, which may seem surprising given our-2 SD criterion for PD-MCI classification. This reflects the heterogeneity of cognitive impairment in PD and the challenges in establishing universally applicable cutoffs. We used a stringent criterion of performance below-2 SD in at least two tests within the same cognitive domain or one test in two or more domains, as suggested by Goldman et al., to enhance specificity in our PD-MCI classification (Goldman et al., 2013, 2015).

Thirdly, while we included age, sex, education level, and total intracranial volume as nuisance variables in our neuroimaging analyses, we did not extensively assess the impact of non-motor symptoms such as depression, apathy, and hallucinations, which are prevalent in PD-MCI. Future studies should consider incorporating these factors to provide a more comprehensive understanding of the relationship between cognitive performance and brain morphology in PD.

Fourthly, our study’s cross-sectional design limits our ability to capture cognitive decline over time. Longitudinal studies would be valuable in elucidating the progression of cognitive impairment and associated brain changes in PD. Lastly, while our neuroimaging analyses revealed correlations between PD-CRS subtest scores and brain morphology, it’s important to note that cognitive functions rely on multiple brain systems. The localizationist approach, while informative, may not fully capture the complexity of brain-behavior relationships in PD-MCI.

Despite these limitations, our study contributes valuable insights into the neural underpinnings of cognitive performance in PD-MCI using the PD-CRS, and highlights the importance of comprehensive neuropsychological assessment in this population. Future research could benefit from larger sample sizes, longitudinal designs, and more advanced analytical approaches to further elucidate the complex relationships between cognitive performance and brain morphology in PD.

## Conclusion

In conclusion, the present study enriches the existent body of literature on the structural neural underpinnings of mild cognitive impairment in Parkinson’s disease (PD-MCI), specifically within H&Y scale stages 1 and 2. Our findings not only corroborate but also extend theoretical frameworks that seek to delineate the anatomical spread of cerebral atrophy in PD. The emergence of cognitive deficits appears to be intrinsically associated with posterior cortical alterations, especially within the parietal and temporal lobes. Regarding the clinical utility of the PD-CRS, our study furnishes compelling evidence of meaningful brain-behavior relationships. Our analyses confirm that PD-CRS scores exhibit significant correlations with multiple cortical regions, thereby lending credence to the hypothesis that measures of cortical volume and thickness in specific regions—such as the superior temporal gyrus, inferior parietal lobule, insula, and precuneus—hold promise as putative surrogate biomarkers for cognitive deterioration in PD.

However, further investigations employing advanced neuroimaging techniques are warranted to elucidate the complex interplay between cognitive task performance and the underlying patterns of neuronal degeneration. Techniques such as whole-brain tractography and Diffusion Tensor Imaging (DTI) could offer valuable insights into the structural and functional integrity of the brain networks involved (Son et al., 2016; Atkinson-Clement et al., 2017). By synthesizing these findings, our study serves as a point of departure for subsequent research to understand the subtle relationship between cortical anatomy and cognitive function in PD, ultimately contributing to improved diagnostic accuracy and therapeutic intervention strategies.

## Data availability statement

The datasets presented in this article are not readily available because the cortical segmentations generated in this study through the utilization of FreeSurfer are accessible to qualified investigators upon reasonable request directed to the corresponding author, PB. This provision particularly applies to research endeavors to deepen our understanding of cerebral alterations associated with Parkinson’s Disease and Mild Cognitive Impairment (PD-MCI). PB has full oversight of all the data collected in this study and bears responsibility for the integrity and analytic accuracy of data. Requests to access the datasets should be directed to PB, pedrobrandao.neurologia@gmail.com.

## Ethics statement

The studies involving humans were approved by the Research Ethics Committee of the Centro Universitário de Brasília (CEUB). The studies were conducted in accordance with the local legislation and institutional requirements. The participants provided their written informed consent to participate in this study.

## Author contributions

PB: Conceptualization, Data curation, Formal analysis, Funding acquisition, Investigation, Methodology, Project administration, Resources, Visualization, Writing – original draft, Writing – review & editing. DP: Conceptualization, Data curation, Formal analysis, Methodology, Writing – original draft, Writing – review & editing. TG: Conceptualization, Funding acquisition, Methodology, Resources, Supervision, Writing – original draft, Writing – review & editing. DB: Conceptualization, Data curation, Formal analysis, Investigation, Methodology, Project administration, Software, Supervision, Visualization, Writing – original draft, Writing – review & editing. FM: Conceptualization, Data curation, Funding acquisition, Methodology, Project administration, Writing – original draft, Writing – review & editing. RT-d-A: Formal analysis, Software, Supervision, Validation, Writing – original draft, Writing – review & editing. BA: Data curation, Formal analysis, Investigation, Methodology, Project administration, Writing – original draft, Writing – review & editing. RM: Supervision, Validation, Visualization, Writing – original draft, Writing – review & editing. MT: Conceptualization, Formal analysis, Funding acquisition, Investigation, Methodology, Project administration, Resources, Software, Supervision, Validation, Visualization, Writing – original draft, Writing – review & editing. FC: Conceptualization, Data curation, Formal analysis, Funding acquisition, Investigation, Methodology, Project administration, Resources, Software, Supervision, Validation, Visualization, Writing – original draft, Writing – review & editing.
